# Effect of surgical microscope and illuminated chopper on anterior chamber temperature

**DOI:** 10.1186/s12886-023-02784-w

**Published:** 2023-01-23

**Authors:** Youngsub Eom, Young Joo Lee, Jong Suk Song, Hyo Myung Kim, Dong Heun Nam

**Affiliations:** 1 Department of Ophthalmology, Ansan Hospital, Korea University College of Medicine, 123, Jeokgeum-Ro, Danwon-Gu, Ansan-Si, Gyeonggi-Do 15355 Republic of Korea; 2 Department of Ophthalmology, Korea University College of Medicine, 73, Goryeodae-ro, Seongbuk-gu, Seoul, 02841 Republic of Korea; 3 Department of Ophthalmology, Emory University School of Medicine, Emory Clinic Building B, 1365B Clifton Road, Atlanta, GA 30322 USA; 4 Department of Ophthalmology, Gil Medical Center, College of Medicine, Gachon University Gil Hospital, 21, Namdong-daero 774beon-gil, Namdong-gu, Incheon, 21565 Republic of Korea

**Keywords:** Microscope, Illuminated chopper, Temperature

## Abstract

**Background:**

To evaluate the effect of the light intensity of the surgical microscope and illuminated chopper on the anterior chamber temperature.

**Study design:**

Experimental study.

**Methods:**

A model eye (Kitaro WetLab System; FCI Ophthalmics, Pembroke, MA, USA) was used in this experimental study. The illuminance of a surgical microscope (Leica M300; Leica Microsystems, Wetzlar, Germany) and illuminated chopper (iChopper NAM-25 GB; Oculight, Korea) with a light source (iVision; Oculight) was measured using an illuminometer. In addition, the temperature in the anterior chamber of the model eye filled with balanced salt solution when using the surgical microscope with a light intensity from level 1 to level 6 and the illuminated chopper at 99% light intensity was measured for 10 min.

**Results:**

The anterior chamber temperature was increased by 0.2, 0.5, 1.0, and 1.4 ℃ when using the surgical microscope at level 3 (10050 lux), 4 (16490 lux), 5 (24900 lux), and 6 (32500 lux), respectively, for 10 min. The illuminated chopper at 99% light intensity (14893 lux) positioned in the anterior chamber increased the anterior chamber temperature by 0.2° C after 10 min, which was equal to the increase in the temperature caused by the surgical microscope at level 3.

**Conclusion:**

The photothermal effect of the illuminated chopper directly positioned in the anterior chamber appeared to be similar to that of a microscope with similar illuminance. Therefore, the illuminated chopper is safe in terms of anterior chamber temperature changes in cataract surgery.

## Background

The use of a surgical microscope is essential in ophthalmic surgeries such as cataract and retinal surgery [1]. The operating microscope has the advantage of facilitating ophthalmic surgery by providing a bright light source to illuminate the ocular surface and intraocular tissue and projecting a magnified image through the lenses. However, retinal phototoxicity due to the bright light source of a surgical microscope as well as endoillumination during pars plana vitrectomy has been reported [2–6].

Recently, cataract surgery using an illuminated chopper, which was modified from an illuminator used in retinal surgery, was introduced to reduce the discomfort of the patient due to the bright light source of the operating microscope [7–10]. Heat from a light source can be transferred by conduction, convection, or radiation (when there is a temperature difference). Heat from the operating microscope is mainly transferred by radiation. On the other hand, heat from the illuminated chopper in the anterior chamber can be transferred by all three methods: conduction, convection, and radiation. However, there is a lack of information on changes in the temperature of the ocular surface and the aqueous humor in the anterior chamber caused by the light source of the operating microscope or illuminated chopper. It is necessary to investigate the safety of the temperature increase caused by the illuminated chopper that continuously emits light in the anterior chamber.

Therefore, the purpose of this study was to determine whether there is a change in the temperature in the anterior chamber according to the brightness of the light emitted from the conventional surgical microscope. In addition, temperature changes in the anterior chamber were investigated using an illuminated chopper, which was inserted directly into the anterior chamber.

## Methods

All experimental procedures for measuring the illuminance and anterior chamber temperature were undertaken in an operating room maintained at 23 ℃. The humidity of the operating room ranged from 49.6% to 52.9%, and the pressure in the room was 1.4 Pa during the experiment.

### Measurement of the illuminance of the surgical microscope and illuminated chopper

The illuminance of a surgical microscope (Leica M300; Leica Microsystems, Wetzlar, Germany) and illuminated chopper (iChopper NAM-25 GB; Oculight, Korea) with a light source (iVision; Oculight) was measured using an illuminometer (TM-205; Tenmars, Taipei, Taiwan). The focal length of the surgical microscope was 20 cm with × 16 magnification, and there were 6 levels of light intensity on the analog scale; the illuminance of the surgical microscope was measured from level 1 to level 6 at 20 cm in the observation plane. The intensity of the light source of the illuminated chopper could be adjusted from 0 to 99%, and the illuminated chopper was placed in the anterior chamber; the illuminance of the illuminated chopper was measured from 10 to 99% in 10% increments (except from 90 to 99%) at a 1 mm distance. The illuminated chopper was fixed at a vertical position, and the light of the illuminated chopper was directed toward the center of the illuminometer sensor. The distance of 1 mm was measured using a digital Vernier caliper (500-144B; Mitutoyo, Tokyo, Japan), and the illuminance was measured while the illuminated chopper and the illuminometer were fixed. The temperature measurement for each condition was repeated three times, and the average value was used.

### Measurement of the anterior chamber temperature when using the surgical microscope or illuminated chopper

A model eye (Kitaro WetLab System; FCI Ophthalmics, Pembroke, MA, USA) was used to investigate anterior chamber temperature changes caused by the surgical microscope or illuminated chopper. The anterior chamber of the model eye was filled with balanced salt solution (BSS) stored at room temperature. The amount of BSS used to fill the anterior chamber was measured.

**Fig. 1:**
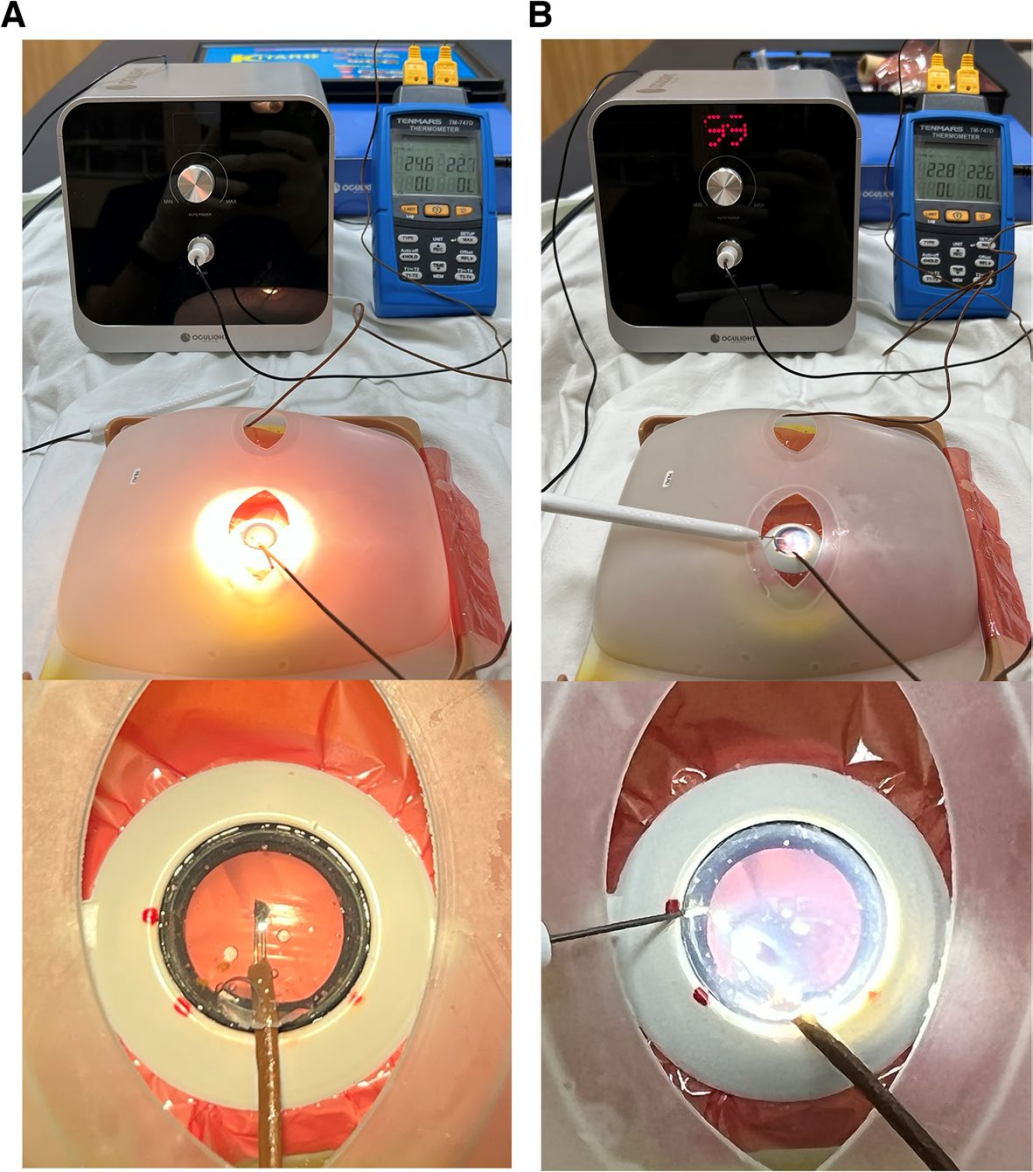
Measurement of the temperature in the anterior chamber of the model eye filled with balanced salt solution (BSS) when using the surgical microscope with a light intensity from level 1 to level 6 (**A**) and the illuminated chopper with 99% light intensity (**B**)

One of two channels of a thermometer (TM-747D; Tenmars, Hsinchu City, Taiwan) was placed in the anterior chamber, and the other channel was placed adjacent to the model eye (Fig. 1). To measure the anterior chamber temperature when using the surgical microscope with a light intensity from level 1 to level 6, the model eye was placed at 20 cm in the observation plane under the microscope (Fig. 1A). To measure the anterior chamber temperature when using the illuminated chopper, the illuminated chopper with 99% light intensity was placed in the anterior chamber of the model eye (Fig. 1B). The anterior chamber temperature and room temperature were measured simultaneously using the two channel of four-channel thermometer at 1 min intervals for 10 min. The temperature measurement for each condition was repeated twice, and the average value was used.

### Statistical analysis

Descriptive statistics were obtained for all experimental data using statistical software (Statistical Package for Social Sciences [SPSS], version 21.0; IBM Corp., Armonk, NY, USA). One-way analysis of variance (ANOVA) was conducted to compare the surgical microscope with a light intensity from level 1 to level 6 and the illuminated chopper at 99% light intensity. Linear regression analyses were conducted to evaluate the relationship between time and anterior chamber temperature or room temperature. *p* < 0.05 was considered statistically significant.

## Results

### Measurement of the illuminance of the surgical microscope and illuminated chopper

**Fig. 2:**
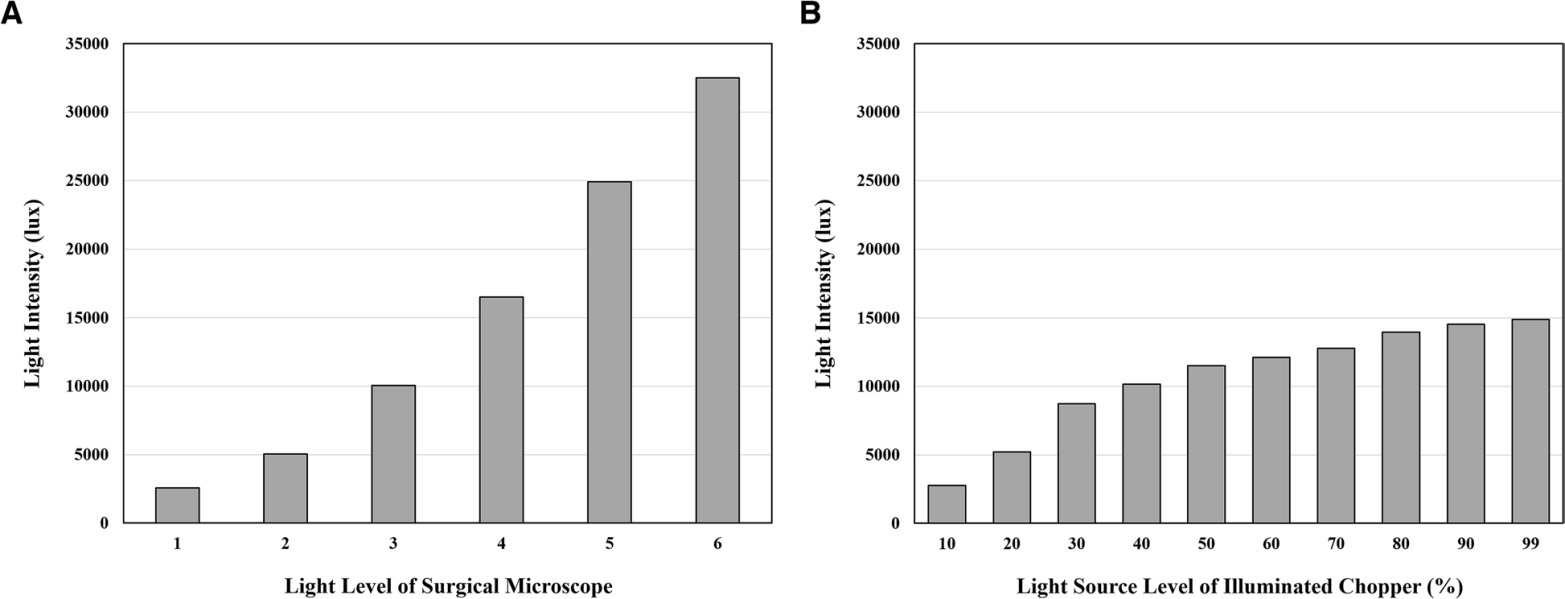
Brightness of the light emitted from the microscope according to the light level of the microscope (**A**) and from the illuminated chopper according to the light source level (**B**)

As the light level of the microscope and illuminated chopper was increased, the brightness of the light emitted from the microscope and illuminated chopper was increased (Fig. 2). The mean (± standard deviation [SD]) illuminance measured 20 cm away from the microscope at level 6 was 32500 ± 250 lux, which was the brightest. The mean (± SD) illuminance measured 1 mm away from the illuminated chopper at 99% light intensity was 14893 ± 45 lux, which was significantly greater than the illuminance measured 20 cm away from the microscope at level 3 (10050 ± 250 lux;* p* < 0.001) and smaller than the illuminance measured 20 cm away from the microscope at level 4 (16490 ± 361 lux; *p* < 0.001).

### Measurement of the anterior chamber temperature when using the surgical microscope or illuminated chopper

**Fig. 3:**
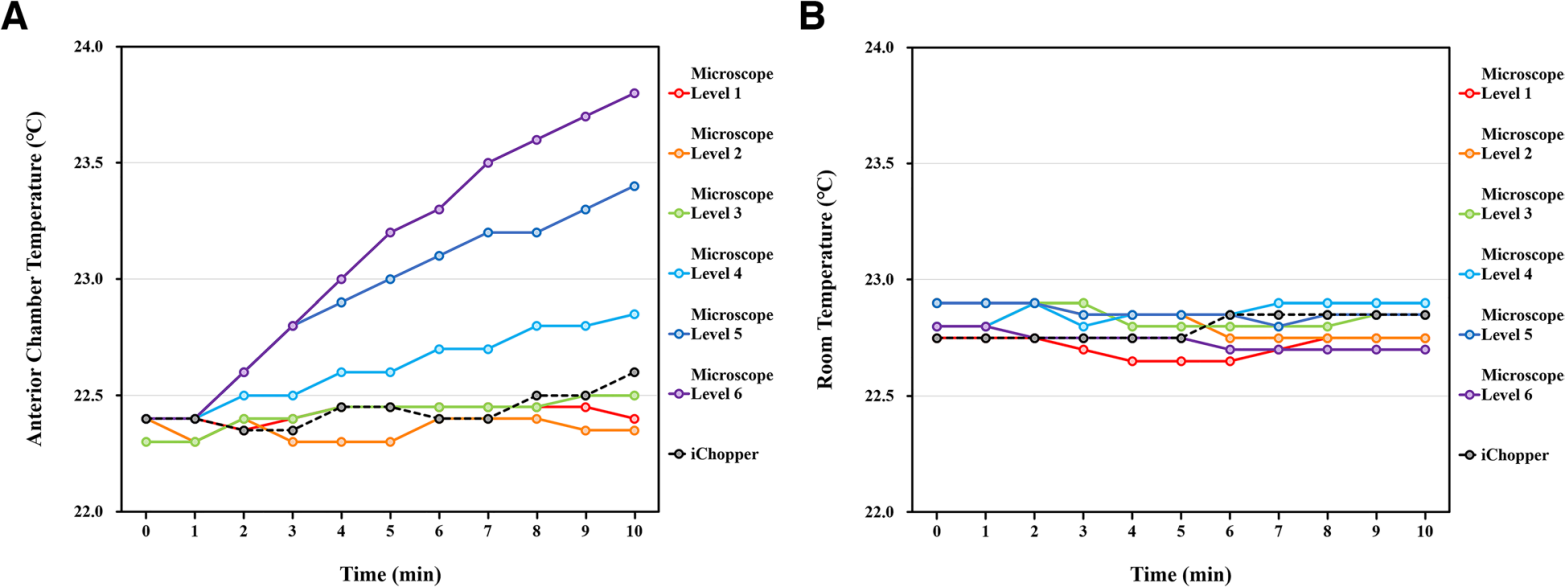
Temperature in the anterior chamber of the model eye filled with balanced salt solution (BSS) (**A**) and room temperature (**B**) when using the surgical microscope with a light intensity from level 1 to level 6 and the illuminated chopper with 99% light intensity for 10 min

The mean amount of BSS filled in the anterior chamber of the model eye was 0.30 ± 0.01 mL. There was no change in the anterior chamber temperature over time when using the microscope at level 1 and 2. However, the anterior chamber temperature was increased when using the illuminated chopper at 99% light intensity and the microscope at level 3, 4, 5, and 6 for 10 min, which was increased by 0.2, 0.2, 0.5, 1.0, and 1.4 ℃, respectively (Fig. 3A). There was no change in the room temperature over time in all experimental conditions (Fig. 3B).

## Discussion

This study investigated temperature changes in the anterior chamber of a model eye caused by a surgical microscope and illuminated chopper. The result of this study showed that as the intensity of the light source of the surgical microscope was increased, the anterior chamber temperature was increased. The anterior chamber temperature was increased by 1.4 ℃ when using the surgical microscope at level 6 (illuminance of 32500 lux) and was increased by 1.0, 0.5, and 0.2 ℃ at level 5 (24900 lux), 4 (16490 lux), and 3 (10050 lux), respectively, for 10 min. In comparison with the surgical microscope placed 20 cm away, the illuminated chopper positioned in the anterior chamber and in direct contact with the aqueous humor was assumed to have a greater thermal effect. However, the illuminated chopper at 99% light intensity (14893 lux) had an illuminance similar to that of the surgical microscope at level 4 and 3 and increased the anterior chamber temperature by 0.2° C after 10 min, which was equal to the increase in the temperature caused by the surgical microscope at level 3. Even with the illuminated chopper positioned in the anterior chamber, the thermal effect of the illuminated chopper appeared to be similar to that of a microscope with similar illuminance. In our institute where this experiment was conducted, the brightness of the surgical microscope is set to a value between 5 and 6 when performing external or intraocular surgery. In addition, the brightness of the light source of the illuminated chopper is set to 40–50% during cataract surgery. Therefore, it seems that surgery using a surgical microscope rather than an illuminated chopper may significantly increase the anterior chamber temperature over time.

The Kitaro WetLab system was introduced for training in several techniques in cataract surgery, and it can also be used to evaluate the efficiency of phacoemulsification [11]. In this study, the Kitaro WetLab system was used to evaluate intraocular temperature changes caused by the surgical microscope and illuminated chopper, and factors that can affect temperature changes inside the human body (e.g., environmental temperature and exposure time) were controlled [12]. Heat gain in the anterior chamber of the human eye from the environment may be associated with conduction in the cornea and aqueous humor, blood perfusion and metabolism in the iris/ciliary body, tear flow and evaporation, and convection in the aqueous humor [13]. However, the heat gain process in the model eye is simpler than that in the human eye, which involves only conduction in the artificial cornea and conduction and convection through BSS.

The artificial cornea of the model eye, which is made from synthetic material, has a central thickness of 500 μm and peripheral thickness of 700 μm to mimic the resistance of the human cornea; however, thermal properties (thermal conductivity and specific heat capacity) are inevitably different from those of the cornea. Thermal conductivity refers to the heat-conducting ability of a material. Therefore, heat conduction occurs at a higher rate in a material with higher thermal conductivity than in a material with lower thermal conductivity. The amount of heat required to increase the temperature of 1 kg of a substance by 1 K is the specific heat capacity [14]. In previous studies, the thermal conductivity and specific heat capacity of porcine cornea were measured as 0.53 W/mK and 3.74 J/gK, respectively [15], whereas those of silicone rubber were 0.2 W/mK and 1.95 J/gK, respectively [14]. Therefore, it seems that heat conduction occurs at a lower rate in silicone rubber than in the cornea. On the other hand, it seems that more heat is required to increase the temperature of the aqueous humor compared with that of BSS as the specific heat capacity of the aqueous humor (4.178 J/gK) [16] was found to be greater than that of BSS (2.45 J/gK) [17].

Previous studies have shown that thermal energy produced by the phaco tip during phacoemulsification could increase the anterior chamber temperature and result in corneal tissue burn and damage [18, 19]. Incision size, ultrasound power, tip design, and aspiration flow rate could affect the corneal incision site temperature [20]. Specifically, irrigation and aspiration flow have been reported to prevent an increase in the incision site temperature [21]. There was no irrigation and aspiration flow in this experimental setting. Therefore, if there is adequate irrigation or aspiration flow, the anterior chamber temperature increase caused by the surgical microscope may be negligible. However, in the case of external eye surgery without irrigation and aspiration flow, although the anterior chamber temperature increase is only 1 ~ 2 ℃, an increase in the intraocular temperature caused by the surgical microscope may occur. In addition, as the temperature continued to increase over time in this experiment, it is thought that the longer the operation time, the greater the anterior chamber temperature increase when using the surgical microscope.

There are some limitations in this study. First, this study was conducted using model eyes filled with room-temperature BSS. As the anterior chamber volume of the model eye is too small to maintain BSS at body temperature in the operating room, the anterior chamber of the model eye was filled with room-temperature BSS to evaluate anterior chamber temperature changes caused by illumination. Therefore, the results of this study may not be applicable to human eyes. Second, although the mechanism of ocular damage by light exposure includes three major forms (photothermal, photomechanical, and photochemical) [22–24], this study only evaluated temperature changes caused by the surgical microscope and illuminated chopper for a given exposure time. In addition, this study did not assess the influence of reflected light or other variables of the light source. The surgical microscope used a halogen bulb as the light source in this study. The findings may not apply to surgical microscopes that use other bulbs such as LED and xenon arc bulbs even though the illuminance of the surgical microscope was measured. Therefore, it is necessary to evaluate the effect of light exposure on ocular tissues when using a surgical microscope or illuminated chopper under various in vivo conditions.

## Conclusions

In conclusion, as the light intensity of the surgical microscope was increased over time, the anterior chamber temperature was increased. In comparison with the surgical microscope, the illuminated chopper directly positioned in the anterior chamber showed a similar photothermal effect. In addition, the illuminance of the illuminated chopper typically used in actual surgery was much lower than that of a surgical microscope. Therefore, the increase in temperature when using the illuminated chopper appeared to be lower than that when using the surgical microscope and was in a range unlikely to cause thermal damage to the eye.

## Data Availability

The datasets during and/or analysed during the current study available from the corresponding author on reasonable request.

## Abbreviations

BSS: Balanced salt solution
ANOVA: Analysis of variance
